# The effect of aged garlic extract on the atherosclerotic process – a randomized double-blind placebo-controlled trial

**DOI:** 10.1186/s12906-020-02932-5

**Published:** 2020-04-29

**Authors:** Martiné Wlosinska, Ann-Christin Nilsson, Joanna Hlebowicz, Anders Hauggaard, Maria Kjellin, Mohammed Fakhro, Sandra Lindstedt

**Affiliations:** 1 Department of Cardiothoracic Surgery and Transplantation, Clinical Sciences, Lund University, Skåne University Hospital, SE-221 85 Lund, Sweden; 2 Department of Cardiology, Clinical Sciences, Lund University, Skåne University Hospital, Lund, Sweden; 3 Department of Radiology, Cardiac Imaging, Skåne Hospital Northwest, Helsingborg, Sweden

**Keywords:** Aged garlic extract, Calcium score, Placebo-controlled, Double-blinded, Blood pressure, Data science, Data mining, CRISP-DM

## Abstract

**Background:**

One of the most serious secondary manifestations of Cardiovascular Disease (CVD) is coronary atherosclerosis. This study aimed to evaluate whether aged garlic extract (AGE) can influence coronary artery calcification (CAC) and to predict the individual effect of AGE using a standard process for data mining (CRISP–DM).

**Method:**

This was a single-center parallel randomized controlled study in a university hospital in Europe. Patients were randomized, in a double-blind manner, through a computer-generated randomization chart. Patients with a Framingham risk score ≥ 10 after CT scan (*n* = 104) were randomized to an intake of placebo or AGE (2400 mg daily) for 1 year.

Main outcome measures were changes in CAC score and secondary outcome measures changes in blood pressure, fasting blood glucose, blood lipids and inflammatory biomarkers.

**Result:**

104 patients were randomized and 46 in the active group and 47 in the placebo group were analyzed. There was a significant (*p* < 0.05) change in CAC progression (OR: 2.95 [1.05–8.27]), blood glucose (OR: 3.1 [1.09–8.85]) and IL-6 (OR 2.56 [1.00–6.53]) in favor of the active group. There was also a significant (*p* = 0.027) decrease in systolic blood pressure in the AGE group, from a mean of 148 (SD: 19) mmHg at 0 months, to 140 (SD: 15) mmHg after 12 months. The AGE Algorithm, at a selected probability cut-off value of 0.5, the accuracy score for CAC progression was 80%, precision score of 79% and recall score 83%. The score for blood pressure was 74% (accuracy, precision and recall). There were no side-effects in either group.

**Conclusions:**

AGE inhibits CAC progression, lowers IL–6, glucose levels and blood pressure in patients at increased risk of cardiovascular events in a European cohort. An algorithm was made and was used to predict with 80% precision which patient will have a significantly reduced CAC progression using AGE. The algorithm could also predict with a 74% precision which patient will have a significant blood pressure lowering effect pressure using AGE.

**Trial registration:**

Clinical trials NCT03860350, retrospectively registered (1/32019).

## Background

Cardiovascular disease (CVD) kills 17.9 million people every year, accounts for 31% of all global deaths, and is considered to be the leading cause of death worldwide [1]. Hypertension and hyperlipidemia are two of the most important risk factors for CVD [1]. Hypertension affects more than 1 billion (one in four) adults worldwide, and about 40% of all premature deaths (under the age of 70 years) worldwide are caused by CVD [1]. One of the most serious secondary manifestations of hypertension and hyperlipidemia is coronary atherosclerosis, which can lead to myocardial infarction. Arteriosclerosis develops over a considerable period of time, which is why primary and secondary prevention have proved to be effective in preventing the development of CVD [2]. Calcified atherosclerotic lesions in the coronary arteries can be measured as coronary artery calcification (CAC) which has become a well-validated prognostic marker of ischemic heart disease (IHD) [3]. The progression of CAC is becoming an acceptable prognostic factor for evaluating the effectiveness of interventions instead of end-points such as myocardial infarction and cardiac disease mortality [4].

The impact of lifestyle and daily diet vary considerably between different countries and continents, and have been shown to affect the development of different kinds of diseases such as diabetes and CVD [5–11]. Lately there has been increased interest and awareness in society regarding the connection between dietary intake and diseases. Primary and secondary prevention using alternative supplements and methods to avoid or to reduce the use of traditional pharmacological drugs have also become popular. One of the reasons for this is that pharmacological drugs with lipid-lowering and blood pressure-lowering effects cause many side-effects that may have a negative impact on the quality of life [12, 13]. Among garlic supplements, aged garlic extract (AGE) has shown high tolerability with a high safety profile, and has a standard dosage of one of the active ingredients S-allylcysteine (SAC) [14–18].

Studies have evaluated the effect of AGE on coronary arteriosclerosis and biomarkers of inflammation in cohorts with low-to-intermediate risk for cardiovascular diseases calculated using the Framingham risk score in non-European cohorts [14, 16, 19, 20]. None of these studies has been conducted on a European cohort with an intermediate-to-high risk of cardiovascular events. Diet differs considerably between different continents and countries. Both diet and ethnicity with genetic variants have been shown to influence the development of cardiovascular disease [21–24]. Thus, a result obtained in one population cannot be predicted to occur in another population. Here, we describe in a randomized placebo-controlled trial of 104 patients with a high risk of cardiovascular events, the effect and tolerability of AGE as a primary but also as an adjunct treatment on blood pressure, blood lipids, and coronary arteriosclerosis and inflammatory biomarkers in a European cohort. The aim was also to create an algorithm, the AGE algorithm, to predict the individual AGE impact on CAC progression and blood pressure.

## Methods

The study was designed as a single-center, parallel, double-blind placebo-controlled randomized study to determine whether AGE can influence the rate of atherosclerosis plaque burden and CAC and inflammatory biomarkers in a European cohort. This study was conducted according to the guidelines laid down in the Declaration of Helsinki and all procedures involving human patients were approved by the local ethical committee DNR 2016/745 (Lund, Sweden). All participants signed a written consent form before entering the study. The study protocol was registered at (https://clinicaltrials.gov/ct2/show/NCT03860350?term=NCT03860350&rank=1) with ClinicalTrials.gov Identifier: NCT03860350. The study was monitored externally by Preventia AB, Sweden, https://www.preventia.se/en/startsida/. The study was conducted according to the CONSORT (Consolidate Standards of Reporting Trials) guidelines and statement [25]. The study was conducted at Skåne University hospital in Lund, Sweden between October 2016 and October 2018.

### Study outcomes

The primary outcome was changes in CAC score after 1 year of placebo or AGE intake. Secondary outcome measurements were changes in blood pressure (diastolic and systolic), fasting blood glucose, blood lipids (total cholesterol, high-density lipoprotein (HDL), low-density lipoprotein (LDL), apolipoprotein A and B and triglycerides) and inflammatory biomarkers (C-reactive protein (CRP) and interleukin–6 (Il–6)).

### Inclusion criteria

Asymptomatic patients aged between 40 and 75 years with a Framingham risk score ≥ 10 [26] were selected and sent for cardiac computed tomography (CT). Patients with a positive CAC score on cardiac CT were included and randomized in the study. The subjects were required to be on stable concomitant medications for at least 4 months prior to randomization and subjects with diabetes were required to have **a** glycated hemoglobin (**H**bA1c) < 8.0, and stable HbA1c level (variation range within 0.5%) for 6 months.

### Exclusion criteria

1) History of myocardial infarction, 2) Symptoms of ischemic heart disease, 3) Proximal CAC, 4) calcium score above 1000 units, 5) Conditions interfering with assessment of coronary calcification (metal clips, bypass patients, intracoronary stents) and drug absorption, 6) hypersensitivity to AGE therapy, 7) any unstable medical disorder, 8) bleeding disorder, 9) stroke, 10) prior life-threatening arrhythmia, 11) resting hypotension (systolic < 90 mmHg) or hypertension (resting blood pressure > 170/110 mmHg), 12) heart failure NYHA class III or IV, 13) history of malignancy within the last 5 years or evidence of active cancer, 14) serum creatinine > 140 μmol/L, 15) triglycerides > 4.0 mmol/L baseline visit, 16) diabetic subjects with HbA1c > 8.0, 17) drug abuse.

### Randomization

A total of 104 patients met the inclusion criteria and were randomized in a double-blind manner, using numbered containers assigned to a computer-generated randomization chart by a study nurse. The patients were randomized to an intake of capsules with 2400 mg AGE daily (two capsules of 600 mg twice daily, Kyolic Reserve formula; Wakunaga of America Co Ltd., *n* = 52) or two placebo capsules twice daily (starch capsules, *n* = 52) for 12 months. All patients receiving AGE supplement received the same dose. Study investigators, i.e. those assessing outcomes and patients, were blinded to treatment allocation. The dose and duration of AGE was determined based on prior publications [16, 19, 20].

### Clinical evaluation

Medical evaluation including medical history, cardiovascular risk factors, prescribed medications, smoking and alcohol intake was performed at 0, 4, 8 and 12 months. In addition, blood pressure measurement, body mass index, ECG measurements and assessment of patients’ compliance with medication were recorded. Blood pressure was measured after 10 min’ rest in a comfortable supine position by an automatic blood pressure monitor (OMRON Automatic Blood Pressure Monitor Model M6 Comfort IT).

### Blood samples

Blood samples were collected and analyzed using standard techniques. The following analyses were made: C-reactive protein (CRP), interleukin–6 (Il–6) fasting blood glucose, blood lipids (total cholesterol, high-density lipoprotein [HDL], low-density lipoprotein [LDL], apolipoprotein A and B and triglycerides).

### CAC measurements

Patients underwent cardiac CT with a 128-multidetector computed tomography scanner, SOMATOM Definition AS+ with Stellar detector by Siemens. Electrocardiographic triggering was performed at 70% of the R-R interval. The coronary arteries were imaged in sequential mode with 3.0 mm (Acq. 32 × 1.2 mm) axial slices. Measurement of Agatston calcium score was performed with software, syngo.via, by Siemens. CAC score measurements were performed on non-contrast studies by an experienced reader blinded to the patient and clinical information. CAC was defined as a plaque of at least three contiguous pixels (area 1.02 mm^2^) with a density of > 130 Hounsfield units. The lesion score was calculated by multiplying the lesion area by a density factor derived from the maximal Hounsfield unit within this area, as described by Agatston et al. [10]. The density factor was recorded in the following manner: 1 for lesions with peak attenuation of 130–199, 2 for lesions with peak attenuation of 200–299, 3 for lesions with peak attenuation of 300–399, and 4 for lesions with peak attenuation of > 400. Total calcium score was determined by totalling individual lesion scores from each of the four main coronary arteries (left main coronary, left anterior descending coronary, left circumflex coronary, and right coronary arteries). Cardiac CT was performed at 0 and 12 months.

### Statistical analyses

A power analysis was based on previously published studies evaluating the effect of garlic and supplements on coronary atherosclerosis, blood pressure, cholesterol and inflammatory biomarkers [19, 20]. All continuous data were presented as a mean value ± SD, and all categorical data were reported as percentages or absolute numbers. Student’s t-test and Chi-squared test were used to assess differences between groups. Comparisons of all parameters between the active therapy and placebo were made by Student’s t-test. Repeated measures ANOVA were performed to test for differences between groups over time. The associations between changes in the two treatment groups (active therapy and placebo) over 12 months for risk factors, including lipid profile, CAC, and CRP were analyzed by logistic regression analyses. These analyses were adjusted for demographics, age, gender, and traditional cardiac risk factors. Odds ratios were calculated for median annual change of CAC progression, change in CRP, and other risk factors. All statistical analyses were performed using SPSS V 19.0 (SPSS Institute, Chicago, IL). The level of significance was set to *p* < 0.05. Odds ratio (OR) was presented for belonging to active group or placebo for lower CAC progression, lower blood glucose and lower inflammatory biomarkers.

### AGE algorithm created using cross industry standard process for data mining (CRISP–DM)

The AGE algorithm was created using a CRISP–DM [27]. A sample of 46 patients who were given AGE supplement treatment were studied with the primary objective of investigating the potential of developing a predictive model. The aim of this was to identify patients for whom CAC and systolic blood pressure progression would benefit from AGE supplement using predictive modelling. The target variable, progression, was created using the measured progression after 12 months’ treatment. By dividing the sample into two groups, where patients with less CAC progression than the median were assigned 1 and the rest were assigned 0, the same methodology was applied for systolic blood pressure, patients with a systolic blood pressure decrease larger than the median were assigned 1 and the rest 0. This meant that those patients who most benefited from the AGE supplement were those assigned the value 1.

Using multivariable methods and logistic regression, two proof-of-concept predictive models were developed and validated using leave-one-out cross validation (LOOCV), one for CAC progression and one for systolic blood pressure. Each model was trained and tested 46 times, resulting in all patients belonging to the training sample 45 times and the test sample once, see Table 1.

**Table 1:** Using multivariable methods, logistic regression, two proof-of-concept predictive models were developed and validated using leave-one-out cross validation (LOOCV), one for CAC progression and one for systolic blood pressure. Each model was trained and tested 46 times, resulting in all patients belonging to the training sample 45 times and the test sample once. An example is seen in the Table

	Patient 1	Patient 2	Patient 3	Patient 45	Patient 46
Model 1	Train	Train	Train	Train	**Test**
Model 2	Train	Train	Train	**Test**	Train
Model 3	Train	Train	Train	Train	Train
Model 45	Train	**Test**	Train	Train	Train
Model 46	**Test**	Train	Train	Train	Train

Various biomarkers were available to use as independent variables in the model, and additional variables were created using basic arithmetic, primarily multiplication. By multiplying variables with other variables and squaring, multiplying the variable with itself, an additional 90 variables were available for the model to use. All analyses were performed in R 3.5.3 using the generalized linear model library for binomial regression and SPSS V19.0 (SPSS Institute, Chicago, IL) using the binary logistic function and forward stepwise (likelihood ratio) variable selection method. The statistics and the AGE algorithm were performed in collaboration with Andreas Timglas at Fanwl Consulting AB.

## Results

**Fig. 1:**
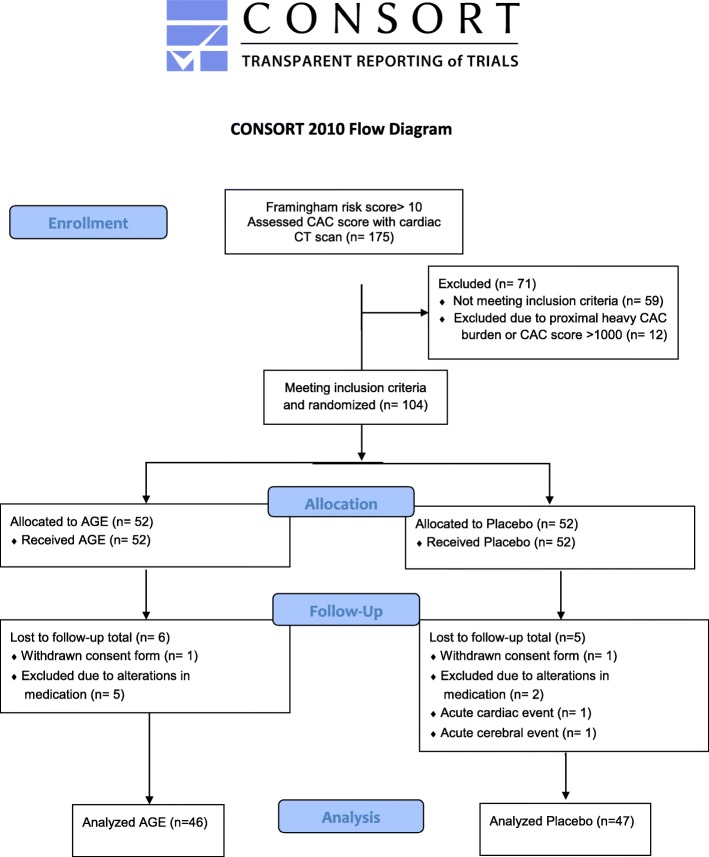
CONSORT statement (consolidated standards of reporting trials) flow chart. Showing demographics and baseline clinical information of the study cohort. Aged garlic extract (AGE), coronary artery calcification (CAC)

A total of 175 patients with a Framingham risk score ≥ 10 were assessed for the study and underwent a cardiac CT scan. Seventy-one patients were excluded after the initial cardiac CT scan: of these 59 did not have a positive CAC score and the remaining had a heavy CAC burden and were sent for further assessment at the cardiology department. In total 104 patients were enrolled and randomized in the study, 11 participants were excluded during the study so consequently 93 patients, 47 in the AGE group and 46 in the placebo group, were analyzed, see CONSORT (Consolidate Standards of Reporting Trials) outlined in Fig. 1. No patient in the study had any adverse reaction to the active therapy that indicated removal from the study.

At baseline there were no significant differences in cardiovascular risk factors calculated using the Framingham risk score. The majority of the patients in the study had medications for hypertension and hypercholesterolemia when they entered the study. Patient demographics are shown in Table 2. There was a significant difference between the AGE group and the placebo group at baseline measurements at 0 months in body mass index (BMI) where the placebo group had a higher BMI than the AGE group. The AGE group on the other hand had a significantly higher CAC score when entering the study. The cholesterol- and LDL-levels were significantly lower in the placebo group than in the AGE group at baseline. Baseline characteristics and absolute values are presented in Table 3.

**Table 2:** Patient demographics at baseline

Variable	AGE		Placebo		
	*n = 46*	*%*	*n = 47*	*%*	*P-value*
Gender (male)	30	(65%)	31	(66%)	0.94
Hypertension	37	(80%)	41	(87%)	0.38
Hypercholesterolemia	24	(52%)	32	(68%)	0.12
Diabetes mellitus	5	(11%)	12	(26%)	0.07
Current smoker	5	(11%)	3	(6%)	0.57
Family history of CVD	33	(72%)	25	(53%)	0.09

**Table 3:** Measurements at baseline, 0 months in absolute values. Coronary artery calcification. (CAC), C reactive protein (CRP). High-density lipoprotein (HDL). Low-density lipoprotein (LDL). Interleukin–6 (Il–6). Apolipoprotein A and B (ApoB/ApoA)

Variable	AGE		Placebo		
	*n = 46*	*SD*	*n = 47*	*SD*	*P-value*
Framingham risk score	23	(7)	21	(7)	0.27
Age (years)	63	(6)	64	(6)	0.95
BMI	27.6	(3.7)	30.0	(4.6)	0.01
CAC	207.3	(237.7)	121.0	(178)	0.05
CRP (mg/L)	2.2	(3.6)	2.2	(3.5)	0.97
Triglycerides (mmol/L)	1.4	(0.7)	1.6	(1.2)	0.21
Cholesterol (mmol/L)	5.2	(1.3)	4.6	(1)	0.03
HDL (mmol/L)	1.6	(0.5)	1.4	(0.4)	0.07
LDL (mmol/L)	3.4	(1.1)	2.9	(0.9)	0.01
Il-6 (ng/L)	4.5	(3.7)	4.4	(2.3)	0.83
ApoB/ApoA	0.7	(0.2)	0.7	(0.2)	0.30
Homocysteine (μmol/L)	13.4	(3.8)	13.2	(3.6)	0.76
Glucose (mmol/L)	6.3	(1.2)	6.6	(1.3)	0.43

### Adjusted multivariable logistic regression analyses

An adjusted multivariable logistic regression analysis was used for equalizing the baseline differences between the placebo and the AGE group. Further calculations using multivariable logistic regression analyses adjusted for age, gender, weight, hypertension, hypercholesterolemia, antiplatelet therapy, heart disease, kidney disease, diabetes, smoking, cancer and alcohol use with median split were used to analyze the data. The results are shown in Table 4.

**Table 4:** Results of a logistic regression with a median split. Coronary artery calcification (CAC), C-reactive protein (CRP). High-density lipoprotein (HDL). Low-density lipoprotein (LDL). Interleukin–6 (Il–6). Apolipoprotein A and B (ApoB/ApoA)

	AGE vs. Placebo		
	*Adjusted OR*	*(95% CI)*	*P-value*
CAC	2.95	(1.05–8.27)	0.040
Glucose (mmol/L)	3.1	(1.09–8.85)	0.034
IL-6 (ng/L)	2.56	(1–6.53)	0.049
Cholesterol (mmol/L)	0.57	(0.23–1.43)	0.228
LDL (mmol/L)	0.62	(0.24–1.58)	0.317
Triglyceride (mmol/L)	1.59	(0.6–4.16)	0.349
HDL (mmol/L)	1.43	(0.55–3.76)	0.463
Homocysteine (μmol/L)	1.18	(0.47–2.96)	0.724
ApoB/ApoA	0.9	(0.34–2.39)	0.840
CRP (mg/L)	1.45	(0.56–3.77)	0.447

### CAC score

The CAC score was measured at 0 and 12 months. Interestingly the adjusted multivariable logistic regression, analyzing the CAC score showed that the probability of belonging to the group with the lowest CAC progression was 2.95 (adjusted OR, 95% CI 1.05–8.27, *p* = 0.040) times higher in the AGE group. CAC is considered to be a progressive disease and a progression in CAC score over time is expected. In the present study all patients, whether in the placebo group or in the AGE supplement group, showed an increase in CAC score over a 12-month period. However, the patients in the AGE supplement group had a decreased progression compared to the placebo group. The placebo group had a significantly increased annular CAC progression of 28% compared to the AGE supplement group (20%).

### P-glucose levels

The probability of belonging to the group with the lowest glucose level was 3.10 (adjusted OR, 95% CI 1.09–8.85, *p* = 0.034) times higher in the AGE group compared to the placebo group.

### Interleukin–6 levels (IL–6)

The probability of belonging to the group with the lowest interleukin–6 (Il–6) was 2.56 (adjusted OR, 95% CI: 1.002–6.53, *p* = 0.049) times higher in the AGE group than in the placebo group. There were no significant differences in the lipid profile, BMI or CRP between the AGE and the placebo groups.

### Blood pressure

Patients’ blood pressure was recorded repeatedly at four time points during the study period. A one-way repeated measure ANOVA analysis was performed to examine the blood pressure. The patients in the AGE group showed significantly (*p* = 0.027) lower systolic blood pressure at the 12 months’ follow-up: 140 (SD: 15) mmHg compared to baseline 148 (SD: 19 mmHg). No difference in systolic blood pressure was seen between 0 and 12 months in the placebo group (baseline 142 (SD 29) mmHg and 14 (SD: 14) mmHg at 12 months) (*p* > 0.996) (Table 5).

**Table 5:** Systolic blood pressure for the AGE and placebo groups, at 0 and 12 months

	Systolic blood pressure				
	0 months		12 months		
Group	*Mean*	*(SD)*	*Mean*	*(SD)*	*P-value*
AGE *(n = 46)*	148	(19)	140	(15)	0.027
Placebo *(n = 47)*	142	(29)	142	(14)	0.996

The diastolic blood pressure in the AGE group was 88 (SD: 9) mmHg at 0 months and 85 (SD: 8) mmHg at 12 months (*p* ≥ 0.06). The diastolic blood pressure in the placebo group was 87 (SD: 9) mmHg at 0 months and 84 (SD: 10) mmHg at 12 months (*p* = 0.049).

### CAC progression – predicted impact of AGE: CRISP–DM

A logistic regression analysis identified six biomarkers as useful for predicting relative change in CAC progression from baseline to the 12-months’ follow up. Using these six biomarkers, a validated, relatively stable predictive model was developed to determine the probability of belonging to the group with the lowest annual change in CAC score. At a selected probability cut-off value of 0.5, the model’s accuracy score was 80%, with a precision score of 79% and recall score of 83% using LOOCV.

Using six biomarkers the following independent variables were created and used to predict CAC progression:

- CAC score at 0 months;
- Plasma concentrations of different lipids;
- Systolic blood pressure and diastolic blood pressure.

### The lowering of systolic blood pressure – predicted impact of AGE: CRISP–DM

A logistic regression analysis identified three biomarkers as useful for predicting relative change in systolic blood pressure progression from baseline to the 12-months’ follow up. Using these three biomarkers, a validated, relatively stable predictive model was developed to determine the probability of belonging to the group with the best effect of AGE on systolic blood pressure, meaning a decrease in the systolic blood pressure after a year of AGE treatment. At a selected probability cut-off value of 0.5, the model’s accuracy score was 74%, with precision score of 74% and recall score of 74% using LOOCV.

Using three biomarkers the following independent variables were created and used to predict systolic blood pressure progression:

- CAC score at 0 months;
- Plasma concentrations of different lipids.

## Discussion

The present study demonstrates that AGE alone reduces the progression of CAC and that the probability of belonging to the group with the lowest CAC progression was almost three times higher in the AGE group than in the placebo group. CAC is considered to be a progressive disease and a progression in CAC score over time is expected. In the present study all patients, whether in the placebo group or in the AGE supplement group, showed an increase in CAC score over a 12-month period. However, the patients in the AGE supplement group had a reduced progression compared to the placebo group. These data are consistent with prior studies documenting the reduced progression of CAC by AGE [14, 16, 18, 20, 28, 29], although all prior studies have been conducted in a non-European population with a low-to-intermediate risk of cardiovascular events. This study is the first of its kind to be performed in a European population with an intermediate-to-high risk of cardiovascular events. The data also show a beneficial effect on inflammation, in line with previous reports [30], with a significant lowering effect on IL-6. The present study also confirms a blood-pressure lowering effect after 12 months of AGE supplement. These results are in line with previous studies showing positive cardiovascular effects of AGE or the combination of AGE with other supplements [19, 20, 31–33]. Interestingly we also found a glucose-lowering effect in the AGE group. Furthermore, in the placebo group there was a positive effect on diastolic blood pressure.

Applying data mining and knowledge discovery as an emerging strategy has increased enormously over the past few years and has been used to predict the outcome of various pharmaceutical and medical treatments [27, 34, 35]. Individuals respond differently to similar treatments presumably since individuals have different biological and genetic determinants of CVD. Biological and genetic determinants of CVD or variations may modulate gene function, affecting certain proteins. These changes could have an impact on the pathogenesis of hypertension and progression of coronary artery disease [36, 37]. Consequently, it is important to be able to customise diverse types of diets, supplements and treatments depending on each individual. We therefore created an algorithm, the AGE algorithm, to predict the individual results of AGE on different outcomes such as lack of CAC progression and blood-pressure lowering effect. We used a CRISP–DM to create the AGE algorithm. The algorithm is based on CAC score, blood lipids and blood pressure at 0 months, and by using these biomarkers we could predict, with 80% precision, which patient will have a significantly reduced CAC progression after 12 months of AGE supplement. With the same type of model we could also predict with a 74% precision which patients will have a significant blood-pressure lowering effect after 12 months of AGE supplement.

The Framingham Risk Score has been validated as a useful tool in the estimation of the 10-year risk of CVD [26]. However, events may still occur among those predicted to be at low risk of CVD [38, 39]. As such, identification of factors associated with CVD events in persons at low risk is imperative. Because of limitations in the Framingham risk score for risk prediction in individuals, much effort has been targeted towards improving identification of persons at risk of coronary events. Coronary artery calcium Agatston score may predict coronary events beyond the Framingham Risk Score risk factors [40]. Increase in CAC scores over time (CAC progression) improves prediction of CVD events. In the present study the patients had no significant differences in Framingham risk score when entering the study, yet their CAC score at baseline was significantly different with a higher CAC score in the AGE group. Since a significant difference at baseline was seen between the AGE and placebo groups a further analysis with a multivariable logistic regression adjusted for cardiovascular risk factors was used for equalizing the baseline differences. Interestingly the adjusted multivariable logistic regression analysis, analyzing the CAC score, showed that the probability of belonging to the group with the lowest CAC progression was 2.95 (adjusted OR, 95% CI 1.05–8.27, *p* = 0.040) times higher in the AGE group. The results from both the present study and prior studies on AGE and CAC score imply that AGE reduces the progression of CAC in both a European and a USA population, which is interesting considering the possible differences in dietary intake between Europeans and US citizens. Statin therapy has also been shown to reduce the rate of CAC progression [2, 41].

Compared to the studies on AGE and CAC progression performed in the past, the present study is by far the largest with approximately 65% more patients than the biggest previous studies [14, 17, 18]. However, in both the present study and in prior studies many of the patients, more or less equally divided between the different cohorts (placebo or AGE groups) were on co-medication with statin therapy, but still the AGE cohort in all studies was shown to result in a slowing CAC progression [14, 16, 19, 20, 28, 31].

A positive effect on IL–6 was observed in the study in favour of the AGE cohort. IL–6 is known to be involved in the inflammatory processes of atherosclerosis [42]. CAC is the result of atherosclerotic disease and can predict future cardiovascular events independent of traditional risk factors [43]. A positive impact on IL–6 might, therefore, partly explain the molecular mechanisms behind the reason why the AGE group showed a lower CAC progression than the placebo group. Other molecular mechanisms behind the beneficial effects of AGE, and other natural products that include garlic (*Allium sativum*), have been studied to some extent. It has been suggested that AGE has a positive impact on vascular endothelial and platelet function with both playing a pivotal role in the etiology of arteriolosclerosis and cardiovascular disease [15, 44–48]. Impaired endothelial function portends an increased risk of cardiovascular disease. Vascular oxidative stress and systemic inflammation play a critical role in the pathogenesis and progression of vascular disease. It is believed that AGE has a positive effect on blood pressure by improving impaired vascular endothelial function, while decreasing the progression of atherosclerotic plaque [49]. IL–6 also stimulates low-grade inflammatory processes and has been proposed to be involved in the pathogenesis causing type 2 diabetes. Mediators of inflammation such as IL–6 among others have been suggested to be involved in these events. IL–6 has, in addition to its immune regulatory actions, been proposed to affect glucose homeostasis and metabolism directly and indirectly by action on skeletal muscle cells, adipocytes, hepatocytes, pancreatic beta-cells, and neuroendocrine cells [50]. Interestingly the patients in the present study also showed lower plasma levels of glucose after 12 months of AGE treatment compared to the placebo group.

### Limitations

There are strengths and limitations of the present study. CAC progression was defined as the absolute change in CAC. Analyses of the progression of CAC are statistically challenging because of a high degree of dependence of the CAC score at baseline and because of inter-scan variability. The cardiac CT scans were all performed on the same CT scan machine by the same staff, and the calculations of the CAC burden were performed by two radiologists. The study was monitored externally during the whole study period, carefully securing that all values were correct and adequate. Although CAC score has become a well-validated prognostic marker of IHD, the absence of CAC does not necessarily exclude obstructive coronary artery disease or the need for revascularization [51]. However, in the present study the focus was not on obstructive stenosis, but on the CAC progression of the coronary arteries over a period of 12 months. CRISP–DM was used to create a proof of concept and presented as the AGE algorithm. Further studies applying the AGE algorithm on bigger cohorts are needed for validation of the algorithm. The serum levels of S-allyl cysteine, to ensure intake, was not measured in the AGE supplement group. However, the patients were taken into clinical check-ups every 3 months and every month the study nurse was in contact with the patient to ensure compliance with the intake of the placebo or AGE supplement.

## Conclusion

The present study demonstrates that AGE alone reduces the progression of CAC and that the probability of belonging to the group with the lowest CAC progression was almost three times higher in the AGE group than in the placebo group. Using data science based on CRISP–DM the AGE algorithm was created. The AGE algorithm was able to predict with 80% precision which patient would have a significant reduction of CAC progression using the AGE supplement. With the same type of model, we could also predict with 74% precision which patient would have a significant blood-pressure lowering effect using the AGE supplement. The data also showed a marked beneficial effect on inflammation, with a significant lowering effect on IL–6. The present study also confirms a blood-pressure lowering effect in a 12-month follow-up period. Furthermore, we showed a glucose-lowering effect in the AGE group.

## Data Availability

The datasets used and/or analyzed during the current study are available from the corresponding author on reasonable request.

## Abbreviations

AGE: Aged garlic extract
CAC: Coronary artery calcification
CRISP-DM: Cross industry standard process for data mining
CRP: C-reactive protein
CT: Computed tomography
CVD: Cardiovascular disease
HbA1c: Glycated hemoglobin
HDL: High-density lipoprotein
IHD: Ischemic heart disease
Il-6: Interleukin–6
LDL: Low-density lipoprotein
LOOCV: Leave-one-out cross validation
OR: Odds ratio
SAC: S-allylcysteine
